# Increased lumbar spinal column laxity due to low‐angle, low‐load cyclic flexion may predispose to acute injury

**DOI:** 10.1002/jsp2.1038

**Published:** 2018-11-28

**Authors:** Nicole C. Gale, Stacey L. Zeigler, Christopher Towler, Sumona Mondal, Kathleen A. Issen, Addisu Mesfin, Arthur J. Michalek, Laurel Kuxhaus

**Affiliations:** ^1^ Department of Mechanical and Aeronautical Engineering Clarkson University Potsdam New York; ^2^ Department of Physical Therapy Clarkson University Potsdam New York; ^3^ Department of Mathematics Clarkson University Potsdam New York; ^4^ Departments of Orthopaedic Surgery and Neurosurgery University of Rochester Rochester New York

**Keywords:** cervine vertebrae, ex‐vivo, lumbar fracture, ring apophysis fracture

## Abstract

Lumbar spinal column laxity contributes to instability, increasing the risk of low back injury and pain. Until the laxity increase due to the cyclic loads of daily living can be quantified, the associated injury risk cannot be predicted clinically. This work cyclically loaded 5‐vertebra lumbar motion segments (7 skeletally‐mature cervine specimens, 5 osteoporotic human cadaver specimens) for 20 000 cycles of low‐load low‐angle (15°) flexion. The normalized neutral zone lengths and slopes of the load‐displacement hysteresis loops showed a similar increase in spinal column laxity across species. The intervertebral kinematics also changes with cyclic loading. Differences in the location and magnitude of surface strain on the vertebral bodies (0.34% ± 0.11% in the cervine specimens, and 3.13% ± 1.69% in the human cadaver specimens) are consistent with expected fracture modes in these populations. Together, these results provide biomechanical evidence of spinal column damage during high‐cycle low‐load low‐angle loading.

## 1 INTRODUCTION

Spinal column stability is achieved via complex interaction between, and neurocontrol of, the flexible spinal column and surrounding musculature.^1^ This stability has a tremendous impact on flexibility, mobility, and ability to carry out activities of daily living. The likelihood of acute lower back injury is also highly dependent on trunk stability. Specifically, increased spinal column laxity (an indicator of decreased stability), when combined with a fatigued neuromuscular system, can lead to increased risk of hyperflexion injury^1^ to both the soft and hard tissue. Spinal column laxity is quantified by the neutral zone length and slope of a load‐displacement curve. Laxity can be increased by repetitive subtraumatic loading (eg, overuse due to sports participation^2^) or occur secondary to acute injury (eg, herniation of the intervertebral discs^3^, ^4^, ^5^ or burst fractures^6^).

Injuries to the hard and soft tissue of the spine can increase spinal column laxity. Vertebral fractures are one such injury and may occur without memorable trauma in older adults with osteoporosis.^7^, ^8^ Vertebral fractures may also occur in younger adults. Lumbar vertebral ring apophysis fractures (RAFs), defined as avulsion type fractures initiating at the posterior aspect of the apophyseal ring, occur without memorable trauma in the skeletally immature spines of adolescent athletes.^4^, ^9^, ^10^ RAFs propagate from posterior to anterior, and therefore may occur during hyperflexion. Given that these fractures can occur without trauma, we believe that both injuries (RAFs and osteoporotic fractures) likely occur during the repetitive loading of activities of daily living (ADLs). These ADLs subject the lumbar spine to approximately 9400 cycles of small flexion/extension movement (<15°) daily,^11^ which may increase laxity of the spine. Furthermore, if the passive laxity of the spine increases, vertebral (bone) strain should increase as the number of cycles of movement increases, increasing the risk of injury. Injuries that change intervertebral disc (IVD) behavior can also affect the laxity of the spine. From ex‐vivo work, it is known that in IVDs, increased laxity is indicated by increased neutral zone length and decreased neutral zone slope after cyclic flexion.^12^

Preventing injuries secondary to increased spinal column laxity is challenging. Yet, specific clinical recommendations for a recovery period following high‐cycle loading are unknown.^13^ The absence of a mechanistic understanding of the link between activities of daily living and increased laxity throughout the day hampers clinical practice. Towards our long‐term goal of providing evidence‐based recommendations for injury prevention secondary to increased laxity, our immediate goal is to quantify the increase in spinal laxity (as evident from a change in neutral zone length) due to low‐angle cyclic flexion loading similar to ADLs. This will ultimately enable identification of the dose of high‐cycle low‐load physical activity that may predispose to acute injury.

We hypothesized that application of high‐cycle non‐traumatic motion to an ex‐vivo model (absent of bone remodeling) would result in biomechanical evidence of increased spinal laxity including increased neutral zone length, decreased neutral zone slope, and changes in both kinematics and bone surface strains. It was further hypothesized that these changes would be more pronounced in adolescent spines than in elderly spines, given that the elderly spines have reduced flexibility due to their increased fibrosity, reduced disc height, and pathological bony changes (eg, osteophytes).

## 2 METHODS

We tested our hypotheses by applying low‐load low‐angle cyclic flexion to adolescent and elderly 5‐vertebra motion segments. Load‐displacement curves, intervertebral kinematics, and surface strain were measured.

### 2.1 Specimen selection

Seven adolescent cervine (2 male, 5 female; 2.4 ± 0.2 years, BMD 1.13 ± 0.20 g/cm^2^) and five elderly human cadaver (1 male, 4 female; 64.2 ± 4.8 years, BMD of 0.91 ± 0.24 g/cm^2^) fresh‐frozen 5‐vertebra motion segments (L1‐L5) were thawed at room temperature (in a sealed, humidified environment), then prepared^14^, ^15^ by careful dissection, preserving the intervertebral ligamentous structures, capsular ligaments, and the posterior longitudinal ligament. Given the scarcity of adolescent human cadaver spines, cervine (white tailed deer, *O. virginianus*) specimens were selected because they have similar anatomy,^16^, ^17^ a similar degree of skeletal maturity, and similar range of motion^14^, ^17^, ^18^ to adolescent humans. (Note that quadruped spines are appropriate models for biomechanical studies of the spine.^19^) The specimens were absent of bony pathologies and had incompletely fused ring apophyses as confirmed with X‐rays (Siemens ICONOS R200, Siemens Corporation, Munich, Germany). The elderly human cadaver specimens exhibited degeneration typical of low BMD; four specimens had visually apparent osteophytes, as confirmed with radiographs, and some degree of disc degeneration. Each motion segment was potted in Bondo (Auto Body Filler, 3M, St. Paul, MN), submerging the cranial half of L1 and the caudal half of L5 to create endcaps to interface with the load frame.^14^ To permit kinematic data collection via motion capture, at least four 4 mm hemispherical retroreflective markers were fixed to the anterolateral aspect of the L2, L3, and L4 with cyanoacrylate adhesive; additional markers were affixed to the endcaps of the fixture. To facilitate the measurement of surface strain via digital image correlation (DIC), matte black paint (Krylon Indoor/Outdoor, Cleveland, OH) was applied to the surface of the specimen with a white (Liquitex acrylic, titanium white, single pigment, Piscataway, NJ) speckle pattern; empirical optimization, via pilot studies, of the painting technique enabled sufficient correlation of the DIC images. Specimen preparation and testing occurred at room temperature (~22°C) and specimens were kept moist with physiologic saline spray (0.159 mol/L) applied at least every 10 minutes throughout preparation and testing. Differences in motion segment behavior when tested in a saline bath, compared to that when tested in air, have been found^20^; however, immersion in a saline bath would have interfered with our measurements of surface strain.

### 2.2 Eccentric cyclic loading

The potted motion segments were placed in a pinned‐end fixture in a load frame (Instron 1331, Norwood, MA) with an eccentricity of 10 mm (see Figure 1 in Corbiere et al.^15^) such that axial compression by the load frame produced combined axial compression and flexion of the motion segment. Specimens were cyclically loaded in displacement control to 20 000 cycles at 0.5 Hz (a ramp waveform) between the neutral position and the displacement corresponding to an initial 15° of flexion (range of absolute displacements: 16 to 23 mm for the cervine cadaver specimens; 14 to 19 mm for the human cadaver specimens). The flexion angle was measured by the regional centroid angle^21^ using a 4‐camera motion capture system (Oqus 500, Qualisys Inc., Göteborg, Sweden) at 120 Hz. This simulates a common ADL movement component of bending forward,^11^ and represents approximately twice the number of cycles per day of small flexion motions.^11^ Periodically during loading, cycling was slowed to 0.01 Hz for five cycles to permit image capture (described below); the fifth cycle was analyzed. These witness tests occurred after cycles 0, 1000, 3000, 6000, 10 000, and 19 995. Load‐displacement data were recorded at 50 Hz.

**Figure 1:**
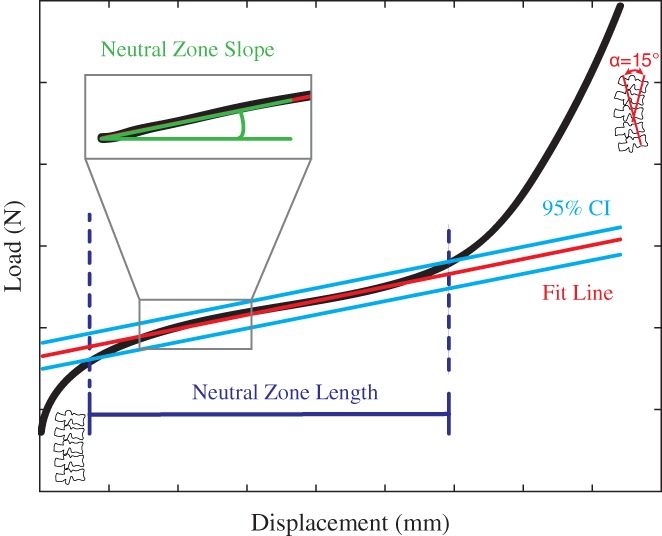
Schematic of neutral zone, neutral zone length, and neutral zone slope

### 2.3 Data collection and analysis methods

Radiographs of all specimens after cyclic loading were interpreted by a collaborating clinician. Load‐displacement data were filtered with a fourth‐order Butterworth filter with a cutoff frequency of 0.01 Hz. The hysteresis loop area of the 5th load‐displacement trace of each witness cycle was computed, as described previously by Corbiere et al.,^15^ using custom MATLAB code. The neutral zone (Figure 1) of the loading trace was also computed using custom MATLAB code (The MathWorks, Natick, MA). Briefly, a line was fit to a window of 1000 data points, centered around an initial point which visually appeared to be within the neutral zone, as selected by the user. (From a practical standpoint, this was easy to visualize as a point in the mid‐range of the constant‐slope middle region of the load‐displacement trace.) A 95% confidence interval of the data points was computed, and a line fit to all data points within this interval. This process of computing a 95% confidence interval and fitting a line to all data points in the interval was iterated to convergence, which was less than 10 times for all cases. Convergence was defined by stabilization of the number of data points assigned to the neutral zone between successive iterations. The neutral zone length was defined by the portion of the data trace between intersections of the load‐displacement data with the ±95% confidence interval. The neutral zone slope was computed using *polyfit* (order one) in MATLAB. To compare across specimens, the neutral zone length and slope for each specimen was normalized by its own neutral zone length and slope at cycle 5. Due to technical complications, one cervine specimen was only loaded to 15 125 cycles and data during witness tests were collected at 5 Hz. Another specimen pulled out of the potting material during testing, and was re‐potted before testing resumed.

In addition to our measurements of load‐displacement data, we measured intervertebral angles to visualize the kinematic effects of laxity changes and surface strain to visualize any changes in surface strain distribution during cycling. During the witness tests, intervertebral angles (IVAs) between both L3‐L4 and L4‐L5 were computed using the QTM software (Qualisys Inc., Göteborg, Sweden). During the witness tests, 3D DIC was used to calculate surface strain accumulated relative to a reference image from cycle 5; strains in the neutral position are relative to cycle 5 neutral position (similarly, strains in the flexed position are relative to cycle 5 flexed position). The 3D DIC system consisted of 2 cameras (Scorpion IEEE‐1394, Point Gray Research, Inc., Richmond BC, Canada) and Vic3D 7 (Correlated Solutions, Inc., Columbia, SC) software. Data were collected at approximately 4 Hz. During data processing, the DIC subset size was approximately a 3 mm square.

### 2.4 Statistical analyses

After verifying assumptions, separate one‐way repeated measures ANOVAs were performed in SPSS (Version 24.0, IBM, Armonk, NY) with a between‐subjects factor of species and within‐subjects factor of hysteresis loop area, neutral zone length, or neutral zone slope. This conservative statistical approach was chosen to be consistent with our hypotheses; that is, we did not test to see if neutral zone length, neutral zone slope, and hysteresis area were different from each other (eg, a multivariate repeated measures anova) because such comparisons would have reduced the overall statistical power of our analysis, and would not have produced meaningful results. For example, the finding that neutral zone length is different from neutral zone slope would be meaningless.) A test of between‐subjects effects was used to determine statistical difference between species. A multivariate test using a Wilks' Lambda distribution and pairwise comparisons was used to see the effect of cycles. Statistical significance was set at α = 0.05.

## 3 RESULTS

The post‐test radiographs reveal no vertebral body fracture in any specimens. However, some elderly human cadaver specimens do show fracture of osteophytes after loading, indicating super‐physiologic flexion; that is, these motion segments are unlikely to have experienced 15° of flexion in vivo.

**Figure 2:**
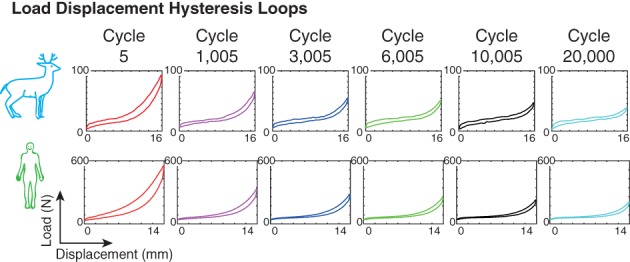
Load–displacement hysteresis loops for representative specimens

**Figure 3:**
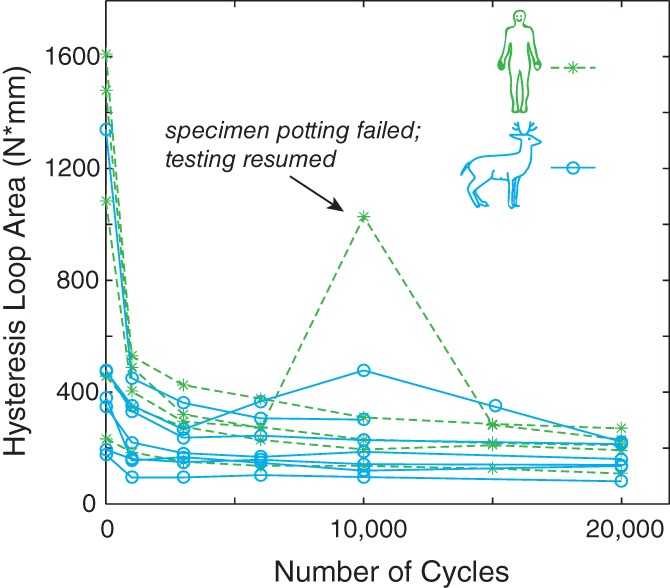
Hysteresis loop area for all specimens; the arrow indicates the specimen in which the potting material failed during the test. Note that this specimen's hysteresis loop area returned to its previous trend when cyclic loading resumed

Figure 2 shows an example of load‐displacement traces; additional figures are available in the Appendix S1. The load‐displacement hysteresis loop area decreased quickly at first, then decayed gradually for all specimens (Figure 3), consistent with our previous results.^15^ This decrease represents the cumulative effect of all changes to the motion segment, including disc relaxation, and is consistent with our previous work that created RAFs in cervine motion segments under similar loading conditions.^15^ The load‐displacement hysteresis loops showed no signs of backlash (which would indicate specimen loosening from the potting material), indicating that loosening did not occur. (This implies that the specimen that pulled out during testing loosened quickly, between witness cycles.) No statistically significant differences between hysteresis loop area and species were detected (*P* = 0.216). There was a statistically significant difference between cycles for the hysteresis loop area (*P* = 0.000): cycle 0 is significantly different from all other cycles, and cycle 20 000 is significantly different from the earlier cycles (Table 1). The peak load of each hysteresis loop also decreased as the number of cycles increased.

**Table 1:** P‐values from statistical comparisons between cycles

Hyesteresis loop area					
	1005	3005	6005	10 005	20 000
5	0.000^a^	0.000^a^	0.000^a^	0.000^a^	0.000^a^
1005		0.011^a^	0.014^a^	1.000	0.000^a^
3005			1.000	1.000	0.000^a^
6005				1.000	0.109
10 005					1.000
Neutral zone length					
	1005	3005	6005	10 005	20 000
5	1.000	0.445	0.230	0.145	0.013^a^
1005		0.650	0.107	0.075	0.003^a^
3005			0.328	0.161	0.027^a^
6005				0.513	0.268
10 005					1.000
Neutral zone slope					
	1005	3005	6005	10 005	20 000
5	0.000^a^	0.000^a^	0.000^a^	0.000^a^	0.000^a^
1005		1.000	1.000	1.000	1.000
3005			1.000	1.000	1.000
6005				1.000	1.000
10 005					1.000

a A significant difference.

**Figure 4:**
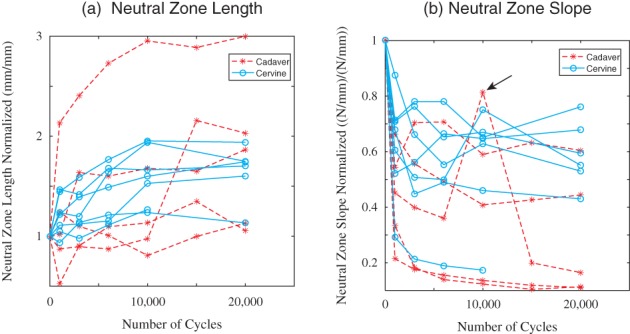
Normalized neutral zone length and slopes for all specimens. The length of the neutral zone generally increases with increasing cycles, and the slope decreases

The normalized neutral zone length increased considerably with increasing number of cycles, and the slope decreases (Figure 4). The normalized neutral zone length increased by 0.65 ± 0.27 (adolescent cervine) and 0.82 ± 0.79 (elderly human cadaver). The normalized neutral zone slope decreased by 0.41 ± 0.12 (adolescent cervine) and 0.71 ± 0.22 (elderly human cadaver). These trends were consistent across all specimens regardless of age and indicate an increase in spinal column laxity. The variability was smaller in the cervine specimens (a more homogeneous population). No significant differences between normalized neutral zone length (*P* = 0.897) or normalized neutral zone slope (*P* = 0.084) and species were detected. There was a significant difference between cycles for the neutral zone length (*P* = 0.03) and neutral zone slope (*P* = 0.00). The neutral zone length at cycle 20 000 was significantly different from the earliest cycles, and the neutral zone slope at cycle 5 was significantly different from all other cycles (see Table 1.)

**Figure 5:**
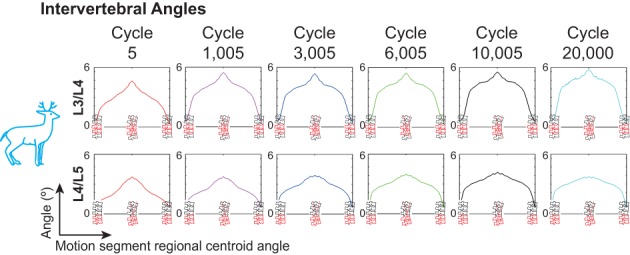
Intervertebral angles for a representative cervine specimen. Note the change in inflection of the later cycles compared to the earlier cycles

The intervertebral angles changed qualitatively with increasing cycles (Figure 5). For example, the total range of motion in L3/L4 increased, while that of L4/L5 stayed approximately constant throughout cyclic loading. The rate of change of the angle (the slope of the angle plots) also changed with increasing cycles. The IVAs from the human specimens were of insufficient quality for analysis; the moist environment created marker visibility problems.

**Figure 6:**
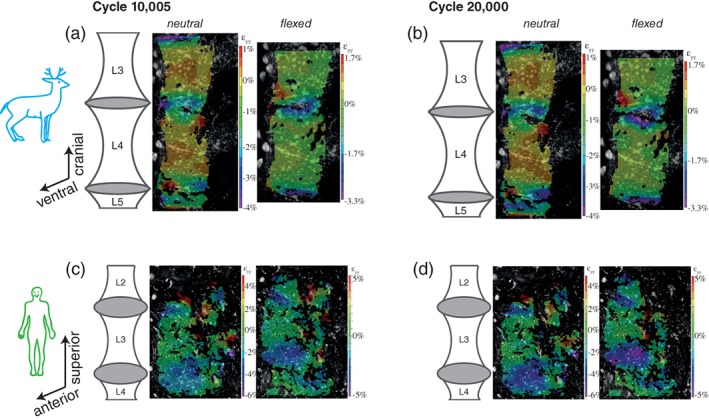
Axial surface strain on the anterolateral surface of representative specimens relative to cycle 5

The strain maps (Figure 6) show the axial surface strain with respect to the 5th cycle. For example, the strain map for 20 000 cycles “flexed” shows axial surface strain due to the change in deformation between the most flexed image from cycle 5 and the most flexed image from cycle 20 000. Similarly, the “neutral” strain map reflected change in deformation between the most neutral position image from cycle 5 and that from 20 000. The surface strain distributions vary between species. In the cervine specimens, (Figure 6, top row), the highest local compressive strains (blue colors) at peak flexion are typically in and around the IVD, including the ring apophyses, as expected. Note the increased relative compressive strain (blue and purple colors) in the L3/L4 IVD in the flexed images. In contrast, the human specimens showed localized strain in areas not adjacent to the IVDs (Figure 6 bottom row; darker colors appear not only near the IVDs.) Comparing the images at 20 000 cycles to those at 10 000 cycles (Figure 6A vs Figure 6B, and Figure 6C vs Figure 6D) showed an increase of the magnitude of strain for both species. Furthermore, there is compressive strain on the disc surfaces by 10 000 cycles (Figure 6B and D). Note that these surface strain measurements were based on the paint applied to the surface of our specimens. Given that the anterior longitudinal ligament was not removed, our measured surface strains may represent apparent strain in the ligament (not the bone.) Additional surface strain images are in the Appendix S1.

Using digital image correlations (distinct from Figure 6) referenced to the undeformed images at peak flexion, the most strain is accumulated in the intervertebral discs, with compressive strains of 6.69 ± 1.95% (cervine) and 5.46 ± 1.68% (human cadaver). The bone had a maximum local compressive axial strain of approximately 0.34 ± 0.11% (cervine) and 3.13 ± 1.69% (human cadaver). Areas of tensile strain were visible in the bone: 0.21 ± 0.11% (cervine) and 0.74 ± 0.66% (human cadaver). These tensile regions in the posterior‐most visible region of the vertebrae suggest similarity in bone behavior to our previous work that generated RAFs in cervine specimens under similar loading conditions. 15 The areas of localized strain in the mid‐vertebral body of the human cadaver specimens are consistent with fracture formation trends in osteoporotic vertebrae.^8^

## 4 DISCUSSION

Our analyses point to a change in lumbar spinal column laxity with increased cycles of low‐load low‐angle flexion. The hysteresis loop area vs cycle (Figure 3) demonstrates similar mechanical behavior between species. The hysteresis loop area of the first witness test of the human cadaver specimens is visually, although not significantly, much larger than that of the cervine specimens. That is, the dissipated energy is relatively higher in the beginning of the test for human cadaver specimens, which is expected due to their much higher volume of viscoelastic (disc) material. It is of particular note that there is no significant difference between cycle 6005 and the additional cycles (as indicated in Table 1), indicating that the viscoelastic system (spinal column) has reached steady‐state behavior.

With cycling, the neutral zone lengths increase, and slope decreases, across all specimens, indicating increased laxity of the lumbar spine motion segment. The adolescent cervine and elderly human cadaver specimens trend similarly during cyclic loading, *despite differences in age, species, and condition* (intact adolescent cervine vs osteoporotic human cadaver specimens.) The significant changes in hysteresis loop area and neutral zone slope between cycle 5 and the subsequent cycles likely reflects disc and other soft tissue relaxation. The significant difference between both hysteresis loop area and neutral zone length at cycle 20 000 compared to the earliest cycles (Table 1) indicates an increase in laxity due to cyclic loading and may indicate a predisposition to injury.

Compressive strain on the anterior side of the discs (Figure 6, cycles 10 005 and 20 000) in the most neutral position image relative to cycle 5 is indicative of wedging of the discs, implying that the anatomical neutral position of the motion segment changes as the number of cycles increases.^22^, ^23^ This modified anatomical neutral state deforms^24^ the nucleus pulposus posteriorly relative to the first cycle of loading, and is consistent with existing literature reports that showed similar deformation at 10 000 or fewer cycles.^25^, ^26^ This deformation alters the center of rotation between the adjacent two vertebrae and also increases lumbar disc herniation risk, and may explain the observed changes in intervertebral kinematics (Figure 5). These changes, in addition to the increase in neutral zone length, confirm the hypothesis that motion segment laxity increases after repeated low‐load low‐angle cyclic loading. Clinically, this suggests that even 10 005 cycles of low‐angle low‐load motion without a recovery period may predispose a lumbar spine to acute injury such as IVD herniation or vertebral fracture. Therefore, a recovery period should occur between repetitive flexion and heavy load bearing activities; future work can identify the necessary period.

The magnitudes of the surface strain are nonuniform, as is expected given the nonuniform geometry and material properties of a vertebral motion segment. Initially, the surface strains are within the range of those experienced during daily activities reported on other bones,^27^ but after cycling, approach previously‐reported bone failure strains.^28^ In particular, the higher anterior‐lateral bone strain in the human cadaver specimens (3.13% ± 1.69%) may be due to the degenerated bone related to lower BMD (and bone mineral content), which is associated with decreased fatigue life of vertebrae.^29^ Due to spinal anatomy, particularly in the cervine specimens, the posterior surface of the ring apophysis was not visible, precluding detection of RAF initiation using surface strain measurements. Although the measurements of anterior surface strains clearly change with increased cycling, recall that the anterior longitudinal ligaments were intact, and the periosteum was removed only when feasible. Therefore, the paint to enable 3D‐DIC was applied over these structures and measurements of surface strain over the anterior longitudinal ligaments may vary slightly from that of the cortical bone. However, given the similarity of the present results to our previous work^15^ that created confirmed RAFs in cervine specimens following cyclic loading and monotonic compression, the present specimens may have, or be predisposed to, formation of these fractures. This predisposition is consistent with another report of endplate separation in cyclically loaded porcine functional spinal units.^30^

Despite limitations of moderate sample size, heterogeneity of specimen populations, and the extended ex‐vivo testing protocol at room temperature, the presence of biomechanical changes following cyclic loading was still evident. Despite the absence of a saline bath, the steady‐state mechanical response indicates that a uniform hydration state was maintained throughout the test.

Both external loads and internal factors affect bending stresses, and therefore injury, to the spine.^31^ Small increases in laxity create more flexibility, similar to warm up activities recommended before vigorous exercise that, in essence, mechanically precondition ligaments and tendons for optimal performance. However, a joint with dramatically increased laxity is more difficult for the neuromuscular system to control, particularly when neural and muscular fatigue occur (such as after repeated cycles of loading.) Rice and McNair^32^ suggested that increased joint laxity may alter the activation of somatosensory receptors, altering neural activities within the spinal reflex pathway by facilitating group I nonreciprocal inhibition of the muscles; in essence, creating a more challenging control problem for the neuromuscular system. Of particular relevance to the spine, it is well‐established from human cadaver studies that spinal column laxity changes diurnally,^33^ increasing lumbar range of motion by over 12° throughout the course of a day. For example, with high‐repetition loading and fatigue, reflex reaction time increases and the maximal force production of fast‐twitch muscles decreases, increasing injury risk as the neuromuscular system is challenged to control a more lax spinal column. A clinical intervention to increase spinal reflex excitability has been suggested to overcome the effects of increased laxity to treat chronic ankle instability.^34^ In the case of spinal motion segments, we postulate that recommendations for sufficient recovery time (to counteract a laxity increase due to repeated small movements) may prevent injury.

## 5 CONCLUSION

This work measured the increase in lumbar spinal column laxity due to cyclic flexion‐extension loading alone. Our ex‐vivo model, absent of bone remodeling, represents a worst‐case scenario. The application of 20 000 flexion cycles to 15° causes a change in motion segment behavior, which may predispose it to injury. When coupled with literature suggesting that fatigue loading can create endplate fractures in healthy vertebrae,^29^ ultimately leading to disc degeneration and low back pain, our work motivates the need for additional means to prevent these injuries and also suggests a mechanism for lumbar disc herniation due to compressive strain. Future work can translate our results into specific recommendations for recovery periods following cyclic ADLs to minimize injury risk.

## CONFLICTS OF INTEREST

LK is a founder with equity in Adaptable Ortho Innovations, a company devoted to bringing innovative orthopedic devices to the market. Author AM has received research and fellowship support from Corelink, the LES Society, AO Spine, and OmEGA.

### Author contribution

Authors NCG, SLZ, KAI, AJM, and LK contributed to the study design. NCG carried out the experiments under the direct supervision of LK, AJM, and KAI. CT and SM contributed to data reduction and analysis. AM ensured clinical realism of the study and analysis. All authors contributed to the interpretation of the results and critical revision of this manuscript.

## Supporting information


**APPENDIX S1**
