# Supplementary material for: Increased lumbar spinal column laxity due to low‐angle, low‐load cyclic flexion may predispose to acute injury
Source: JOR Spine. 2018 Nov 28;1(4):e1038. doi: 10.1002/jsp2.1038 (PMC6686791; doi:10.1002/jsp2.1038)
Supplement: Supplementary file 1 — APPENDIX S1 [file JSP2-1-e1038-s001.pdf]

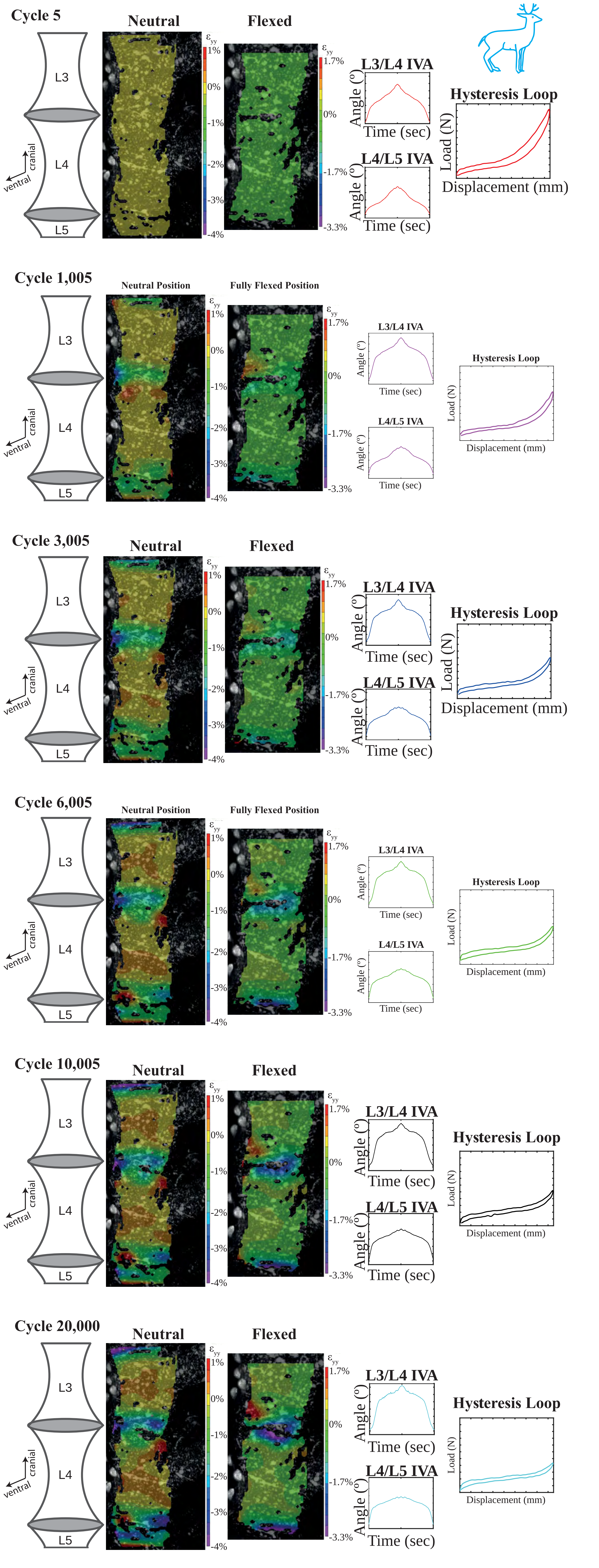
Cycle 5
Neutral
Flexed
L3
L4
L5
cranial
ventral
εyy
1%
0%
-1%
-2%
-3%
-4%
1.7%
0%
-1.7%
-3.3%
L3/L4 IVA
Angle (º)
Time (sec)
L4/L5 IVA
Angle (º)
Time (sec)
Hysteresis Loop
Load (N)
Displacement (mm)
Cycle 1,005
Neutral Position
Fully Flexed Position
L3
L4
L5
cranial
ventral
εyy
1%
0%
-1%
-2%
-3%
-4%
1.7%
0%
-1.7%
-3.3%
L3/L4 IVA
Angle (º)
Time (sec)
L4/L5 IVA
Angle (º)
Time (sec)
Hysteresis Loop
Load (N)
Displacement (mm)
Cycle 3,005
Neutral
Flexed
L3
L4
L5
cranial
ventral
εyy
1%
0%
-1%
-2%
-3%
-4%
1.7%
0%
-1.7%
-3.3%
L3/L4 IVA
Angle (º)
Time (sec)
L4/L5 IVA
Angle (º)
Time (sec)
Hysteresis Loop
Load (N)
Displacement (mm)
Cycle 6,005
Neutral Position
Fully Flexed Position
L3
L4
L5
cranial
ventral
εyy
1%
0%
-1%
-2%
-3%
-4%
1.7%
0%
-1.7%
-3.3%
L3/L4 IVA
Angle (º)
Time (sec)
L4/L5 IVA
Angle (º)
Time (sec)
Hysteresis Loop
Load (N)
Displacement (mm)
Cycle 10,005
Neutral
Flexed
L3
L4
L5
cranial
ventral
εyy
1%
0%
-1%
-2%
-3%
-4%
1.7%
0%
-1.7%
-3.3%
L3/L4 IVA
Angle (º)
Time (sec)
L4/L5 IVA
Angle (º)
Time (sec)
Hysteresis Loop
Load (N)
Displacement (mm)
Cycle 20,000
Neutral
Flexed
L3
L4
L5
cranial
ventral
εyy
1%
0%
-1%
-2%
-3%
-4%
1.7%
0%
-1.7%
-3.3%
L3/L4 IVA
Angle (º)
Time (sec)
L4/L5 IVA
Angle (º)
Time (sec)
Hysteresis Loop
Load (N)
Displacement (mm)

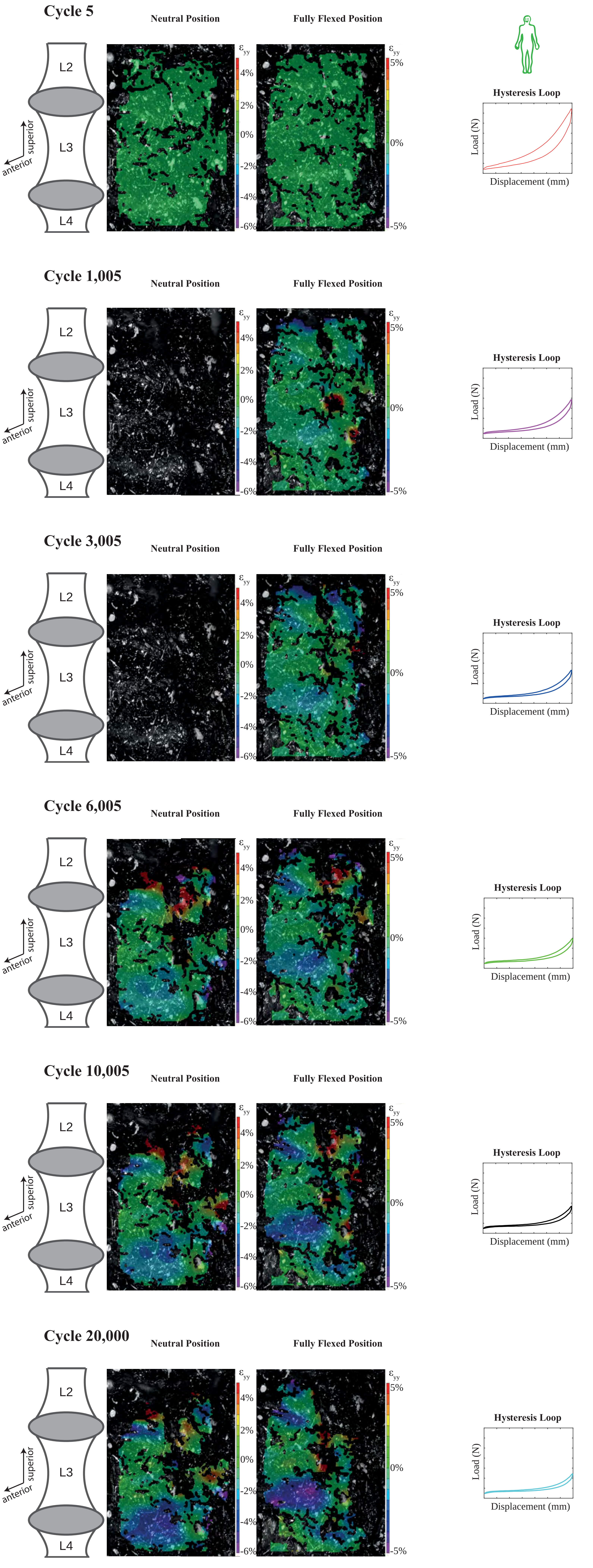

Cycle 5
Neutral Position
Fully Flexed Position
L2
L3
L4
superior
anterior
ε_{yy}
4%
2%
0%
-2%
-4%
-6%
5%
0%
-5%
Hysteresis Loop
Load (N)
Displacement (mm)
Cycle 1,005
Neutral Position
Fully Flexed Position
Hysteresis Loop
Load (N)
Displacement (mm)
Cycle 3,005
Neutral Position
Fully Flexed Position
Hysteresis Loop
Load (N)
Displacement (mm)
Cycle 6,005
Neutral Position
Fully Flexed Position
Hysteresis Loop
Load (N)
Displacement (mm)
Cycle 10,005
Neutral Position
Fully Flexed Position
Hysteresis Loop
Load (N)
Displacement (mm)
Cycle 20,000
Neutral Position
Fully Flexed Position
Hysteresis Loop
Load (N)
Displacement (mm)
